# Management of Infected, Non-Responsive Atopic Dermatitis in a Romanian Center

**DOI:** 10.3390/jcm15135248

**Published:** 2026-07-05

**Authors:** Raluca-Gabriela Miulescu, Ioana Roșca, Alexandru-Neculai Pavel, Ruxandra-Cristina Marin, Andreea Teodora Constantin, Monica Costescu, Elena Poenaru, Daniela Eugenia Popescu, Oana Andreia Coman

**Affiliations:** 1 Faculty of Medicine, “Carol Davila” University of Medicine and Pharmacy, 020021 Bucharest, Romania; miulescuraluca@yahoo.com (R.-G.M.); ioana.rosca@umfcd.ro (I.R.); ruxandra.marin@umfcd.ro (R.-C.M.); andreea.constantin@umfcd.ro (A.T.C.); monica.costescu@umfcd.ro (M.C.); oana.coman@umfcd.ro (O.A.C.); 2 Dermatology Department, Saint Constantin Hospital, 500388 Brasov, Romania; 3 Clinical Hospital of Obstetrics and Gynecology “Prof. Dr. P.Sirbu”, 060251 Bucharest, Romania; 4 Pax Clinic, 020951 Bucharest, Romania; alexnpavel@yahoo.com; 5 FutureMeds, 800001 Galati, Romania; 6 Pediatrics Department, National Institute for Mother and Child Health “Alessandrescu-Rusescu”, 20382 Bucharest, Romania; 7 Dermatology Department, Clinical Hospital for Infectious and Tropical Diseases “Dr. Victor Babes”, 050589 Bucharest, Romania; 8 Department of Obstetrics-Gynecology and Neonatology, “Victor Babeș” University of Medicine and Pharmacy, 300041 Timișoara, Romania

**Keywords:** atopic dermatitis in pediatric patients, skin dysbiosis, *Staphylococcus aureus*, food allergies, microbiota

## Abstract

**Background**: Atopic dermatitis is a chronic inflammatory skin disease in children, frequently associated with skin barrier dysfunction, immune dysregulation, and dysbiosis. Infected, treatment-resistant lesions may increase disease severity and complicate management, particularly in the presence of *Staphylococcus aureus* colonization. **Objectives**: To characterize the microbiological profile of infected, non-responsive pediatric atopic dermatitis, evaluate short-term clinical outcomes following individualized treatment, and identify predictors of disease severity. **Methods**: This observational analytical study included 41 children with atopic dermatitis recruited at Saint Constantin Hospital, Brașov, Romania, between September 2025 and February 2026. Eligible patients fulfilled the Hanifin and Rajka criteria and presented with infected, treatment-resistant lesions. Skin cultures were subjected to an antibiogram and antifungigram. Disease severity was assessed using the Patient-Oriented Eczema Measure (POEM) and SCORAD at baseline, 7 days, and 30 days. Repeated-measures ANOVA, mixed ANOVA, and hierarchical linear regression were used for statistical analysis. **Results**: *Staphylococcus aureus* was the predominant pathogen, followed by other bacterial species. Both POEM and SCORAD scores improved significantly over the 30-day follow-up, with marked improvement after 7 days and further reduction by day 30. Although patients with *S. aureus* colonization and those receiving systemic therapy tended to have higher disease severity, neither factor significantly influenced the trajectory of clinical improvement. Baseline disease severity was the strongest predictor of 30-day POEM and SCORAD outcomes, whereas demographic and perinatal characteristics did not independently predict short-term clinical outcomes. **Conclusions**: Individualized management was associated with significant improvements in clinician-assessed disease severity and patient-reported symptoms during the 30-day follow-up. *Staphylococcus aureus*, particularly methicillin-sensitive *S. aureus* (MSSA), was the most frequently isolated pathogen. Baseline disease severity was the strongest predictor of short-term clinical outcomes, whereas the evaluated demographic and perinatal characteristics did not provide additional predictive value in this cohort. Larger prospective controlled studies are needed to confirm these findings.

## 1. Introduction

Atopic dermatitis (AD) represents a dermatological condition and affects pediatric patients. AD manifests as skin barrier dysfunction and inflammation. Most often, this skin condition is one of the first diseases of the atopic march [1]. Skin barrier dysfunction is caused by disruption of the stratum corneum; this outer layer of the skin is crucial for maintaining hydrophobic properties, rheological properties, and skin pH. The skin of patients suffering from AD presents Th 2 inflammation because of the passage of allergens through the skin. Genetic factors, such as filaggrin mutation, are associated with asthma, food allergy, or AD [2].

The natural pH of the skin is usually acidic (values from 4 to 6). In order to maintain the functions of the skin, it is important to maintain the proper hydration of the epidermis, the integrity of the stratum corneum, and, of course, of the skin pH. So, meanwhile, the proper pH keeps resident flora and protease functioning within normal limits. Therefore, pediatric patients with AD present an increased pH, with the highest value for skin lesions [3].

The skin microbiota are the microorganisms that are resident on our skin, and are represented by bacteria, fungi, viruses and mites; its role is to maintain barrier integrity and immunity. The most important factors that influence skin microbiota are body location and sebum production; other factors include sex, age, ethnicity, hormones, lifestyle, and hygiene [4,5,6]. On the other hand, use of antibiotics and antiseptics reduce pathogens but promote resistance [7]. Commensal microorganisms occupy ecological niches and compete with the pathogens for nutrients and space; they produce antimicrobial substances, especially against *Staphylococcus aureus*. The higher concentration of microorganisms is represented by bacteria (*Actinobacteria*, *Firmicutes*), followed by viruses and fungi (for the last two, it is even higher than in gut microbiota) [8,9]. As for the location of the bacteria, it seems that, on the necks of pediatric patients with AD, there is a reduced presence of Malassezia spp. [10]. Some studies have shown that skin microbiota transplants may attenuate symptoms of AD, which means that these children have also lost the protective role of the commensal microorganism [11].

Skin microbiota development passes through various changes once an infant is born. Recently, a group of authors proposed that exposure to bacterial communities starts in utero [12]. On the other hand, other studies have highlighted that, prior to labor, in uncomplicated pregnancies, there are no live bacteria [13]. In neonates, the changes in the skin barrier function are due to rapid surface colonization. The most important changes in skin barrier appear in the first months of life, and less often after [14]. The skin microbiota changes with age; in neonates aged 0–6 months, the common bacteria are *Staphylococcus*, *Streptococcus*, and *Corynebacterium*; in infants aged 6 months to 1 year old, they are *Prevotella*, *Gemella*, *Cutibacterium*, *Enterococcus*, and the ones mentioned before; and in children aged 2 to 12 years old, they are *Streptococcus*, *Dolosigranulum*, *Gemella*, *Granulicatella*, *Moraxella*, *Haemophilus*, *Neisseria*, and *Rothia* [15].

The microbial diversity is often lower in atopic skin compared to healthy skin, especially in locations affected by AD: the popliteal fossa and antecubital fossa [16].

AD associated with skin infections, with an increase in *Staphylococcus aureus*, was positively correlated with SCORAD score. Also, the severity of the eruption was influenced by the colonization of *Staphylococcus aureus* in lesional skin, and the ability to form biofilms [17,18]. On the other hand, *Staphylococcus Hominis* is negatively correlated with SCORAD score [19].

Despite the recognized association between skin dysbiosis, *Staphylococcus aureus* colonization, and atopic dermatitis severity, evidence regarding the microbiological profile and short-term clinical evolution of pediatric patients with infected, treatment-resistant lesions in routine clinical practice remains limited. Therefore, the primary objective of this study was to characterize the microorganisms isolated from infected, non-responsive atopic dermatitis lesions in a Romanian pediatric cohort. The secondary objectives were to describe the therapeutic management applied in these patients, evaluate changes in disease severity over a 30-day follow-up using POEM and SCORAD scores, and explore whether selected demographic, perinatal, microbiological, and treatment-related factors were associated with clinical outcomes.

## 2. Materials and Methods

### 2.1. Study Design and Patient Selection

This observational analytical study included pediatric patients diagnosed with atopic dermatitis (AD) who were recruited from Saint Constantin Hospital, Brașov, Romania, between September 2025 and February 2026. Consecutive eligible patients presenting during the study period were screened for enrollment. Eligibility was assessed before any study procedures were performed, and only patients fulfilling all inclusion criteria and none of the exclusion criteria were included in the final analytical cohort. Inclusion criteria were patients under 18 years of age, diagnosed with AD of any form, according to the Hanifin and Rajka criteria; pediatric patients with AD, who also had associated infected skin lesions; eruption that was non-responsive according to first lines of treatment in the guidelines. The major Hanifin and Rajka criteria were pruritus, early age of onset, chronic dermatitis, typical morphology and distribution, and personal or family history of atopy; minor criteria included xerosis, pityriasis alba, nipple eczema, hand/foot nonspecific dermatitis, white dermographism, and susceptibility to cutaneous infection.

Exclusion criteria: patients under 18 years of age, with any other form of eczema; other autoimmune skin diseases; infectious diseases; recent use of probiotics or antibiotics in the last 4 weeks; pediatric patients whose caregivers did not respond to all questions. In total, 41 children with non-responsive forms of AD and infected lesions were included in the study. The informed consent form for participation and processing of personal data was signed by the legal representatives of all participants. All enrolled participants completed the scheduled study visits at baseline, 7 days, and 30 days. Consequently, the complete cohort of 41 patients was included in all longitudinal and regression analyses, and no missing outcome data required imputation.

The methods used for the investigation of skin infection included bacteriological culture with antibiotic susceptibility testing (antibiogram) and, when indicated, mycological culture with antifungal susceptibility testing (antifungigram). Skin swab specimens were collected from clinically infected lesions during the baseline visit and before initiation of antimicrobial therapy. Sterile swabs were premoistened with saline solution supplemented with 0.1% Tween 20 and gently rubbed over the affected skin according to the routine sampling procedure. Samples were processed in the hospital microbiology laboratory using standardized microbiological procedures for bacterial identification and antimicrobial susceptibility testing, with antifungal susceptibility testing performed when fungal growth was detected. Microorganisms investigated by culture included *Enterococcus* spp., *Streptococcus* spp., *Staphylococcus epidermidis*, Enterobacteriaceae, *Pseudomonas* spp., *Acinetobacter* spp., and *Candida* spp.

The primary objective of this study was to characterize the microorganisms isolated from infected, non-responsive atopic dermatitis lesions. Secondary objectives included describing the therapeutic management, evaluating changes in disease severity during the 30-day follow-up, and exploring factors associated with clinical outcomes.

### 2.2. Clinical Evaluation

The impact of AD on pediatric patients was assessed using the POEM questionnaire. It quantifies the quality-of-life impairment of the child in the preceding week. Also, the group was divided into 2 smaller groups: younger than 5 years old (the caregiver answered for the child), and over 5 years old (the child answered with the caregiver). The POEM questionnaire has 7 items, covering the following 6 aspects of the impact of eczema on the quality of life, during the past week: itching, sleep disturbance, bleeding eczema, weeping or oozing eczema, cracking and flaking because of eczema, dry or rough skin. The maximum POEM score is 28, with the following degrees of severity: 0–2 clear/almost clear; 3–7 mild; 8–16 moderate; 17–24 severe; 25–28 very severe.

At the same visit, SCORAD was completed to quantify clinician-assessed disease severity. For this score, we used the SCORAD calculator, which includes area involved (body surface area); the intensity of the eczema (for this, a representative area is selected): redness, swelling, oozing/crusting, scratch marks, lichenification, dryness; subjective symptoms: a visual analogue scale is used, for itching and sleeplessness. The 2 questionnaires were completed during the medical visit. The POEM questionnaire was administered in paper format, whereas SCORAD was calculated by the dermatologist using the Scoring Atopic Dermatitis application. Both outcome measures were recorded at every study visit using the same standardized assessment procedures. POEM captured patient- or caregiver-reported symptoms, whereas SCORAD provided a clinician-based evaluation of disease severity, allowing for complementary assessment of clinical response over time.

In order to be able to compare the preterm with term children, the following data was also recorded: sex, age, preterm or term newborn, other associated atopic diseases, cesarean section compared with vaginally delivered children, in vitro fertilization, newborn breastfed or formula.

For this group of pediatric patients, we also noted POEM and SCORAD scores before starting the treatment (visit 0). In order to quantify the efficacy of the therapy, the scores were also calculated during the first visit (after 7 days), and the second visit (after 30 days). At each follow-up visit, patients underwent standardized clinical reassessment, including POEM and SCORAD evaluation, review of disease progression, and treatment adjustment when clinically indicated according to the patient’s response and microbiological findings.

### 2.3. Statistical Analysis

Statistical analyses were performed using IBM SPSS Statistics, version 26 (IBM Corp., Armonk, NY, USA). Continuous variables are presented as mean ± standard deviation (SD) and categorical variables as frequency and percentage. The significance threshold was set at α = 0.05 unless otherwise specified. All analyses were performed using complete-case data, as no participants were lost to follow-up and no missing outcome data were recorded during the study period.

To evaluate the evolution of disease severity over time, one-way repeated measures analyses of variance (ANOVA) were conducted separately for the Patient-Oriented Eczema Measure (POEM) and the SCORing Atopic Dermatitis index (SCORAD), with time (baseline, 1 week, 30 days) as the within-subjects factor. Sphericity was assessed using Mauchly’s test; where the assumption was violated, degrees of freedom were corrected using the Greenhouse–Geisser (G-G) estimate. Post hoc pairwise comparisons between all three time points were performed with Bonferroni correction. Effect sizes are reported as partial eta-squared (η^2^), interpreted as small (≥0.01), medium (≥0.06), or large (≥0.14) according to Cohen’s conventions [20].

To examine whether systemic therapy or Staphylococcus aureus skin colonization status moderated the trajectory of POEM and SCORAD scores over time, two separate mixed ANOVAs were conducted, each incorporating time as the within-subjects factor and one binary variable (systemic therapy yes/no; S. aureus positive/negative) as the between-subjects factor. Because observed (post hoc) power is a deterministic function of the obtained *p* value and adds no information beyond it, it is not used to interpret non-significant findings; instead, interaction effects are reported as partial eta-squared with 95% confidence intervals, and the study is acknowledged as underpowered a priori for these subgroup and interaction analyses.

To identify baseline predictors of disease severity at 30 days, hierarchical linear regression analyses were performed separately for POEM and SCORAD at 30 days (T3) as the dependent variable. Baseline scores (T1) were entered in Block 1 to account for initial disease severity, followed by perinatal and demographic characteristics (age, sex, premature birth, breastfeeding, cesarean section, and in vitro fertilization) in Block 2. This approach, recommended by Vickers and Altman [21], maximizes statistical power while appropriately controlling baseline severity. The change in R^2^ (ΔR^2^) and its significance were used to evaluate the contribution of Block 2 predictors beyond baseline severity alone.

Supplementary mixed ANOVA analyses were conducted to examine whether individual treatment variables (emollients, corticosteroids, antibiotics) and perinatal/demographic characteristics separately moderate POEM and SCORAD trajectories. A Bonferroni correction was applied within each family of tests (α = 0.017 for three treatment comparisons; α = 0.008 for six baseline characteristic comparisons). Given the exploratory nature of these supplementary analyses and the limited sample size (N = 41), all findings are interpreted with caution. Post hoc power analyses were conducted using G*Power 3.1 to estimate sample sizes required for adequately powered future studies.

## 3. Results

### 3.1. Study Population

Forty-one children with atopic dermatitis were included (24 male, 58.5%; median age 12 months (IQR 7–60); mean 39.78 ± 45.64 months). All 41 enrolled participants completed the scheduled follow-up assessments at 7 and 30 days; therefore, all analyses were performed on the full study cohort. The majority were born by cesarean section (34, 82.9%), with 12 (29.3%) born prematurely and 5 (12.2%) conceived via in vitro fertilization. Fifteen children (36.6%) were breastfed as newborns. Baseline POEM and SCORAD scores indicated moderate-to-severe atopic dermatitis (POEM: 15.41 ± 5.11; SCORAD: 45.89 ± 11.51). Full sample characteristics are presented in Table 1.

Regarding treatment, the majority received local antibiotic therapy (34, 82.9%) and emollients (36, 87.8%), while corticosteroids were prescribed in 25 cases (61.0%).

As for topical antibiotics, most of the children received gentamicin (24/34, 70.6%), followed by fusidic acid (20/34, 58.8%), and then mupirocin (19/34, 55.9%). Because of some lesions associated with crusts, these patients were also recommended chlorhexidine solution for disinfection (2/41, 4.9%).

All pediatric patients received emollients, in order to control the microinflammation.

For the inflammatory phenomenon, topical corticosteroids were prescribed: low-potency dermatocorticosteroids, hydrocortisone (11/25, 44.0%); medium-potency betamethasone (9/25, 36.0%), and mometasone (4/25, 16.0%). Another class of drugs recommended for their anti-inflammatory effect was calcineurin inhibitors: pimecrolimus (7/41, 17.1%) and tacrolimus (1/41, 2.4%).

Eleven children (26.8%) required systemic therapy in addition to local treatment: trimetoprim-sulfametoxazole (5, 45.45%); cefuroxime (2, 18.18%); vancomycin (1, 9.09%); gentamicin (1, 9.09%); ceftriaxone (1, 9.09%); cefpodoxime (1, 9.09%).

Skin culture was positive for *Staphylococcus aureus* in 19 patients (46.3%): MSSA (15; 78.9%) or MRSA (4, 21.1%).

Other organisms were identified in 14 patients (34.1%): *Enterobacter cloacae* (1, 7.14%); *Klebsiella oxytoca*, *Staphylococcus aureus*, *MSSA* (1, 7.14%); *Pantoea aglomerans* (2; 14.3%); *Staf hominis* (1; 7.14%); *Staf. Epidermidis* (3, 21.42%); *Enterococcus faecium* (1; 7.14%); *Staf warneri* (3, 21.4%); *Staf sciuri* (1; 7.14%); *Proteus mirabilis* (1; 7.14%); *Streptococcus viridans* (1; 7.14%); *Klebsiella pneumoniae* (1; 7.14%).

### 3.2. Evolution of POEM and SCORAD Scores over 30 Days

Both POEM and SCORAD scores demonstrated significant and progressive improvement across all three time points. Mean POEM scores decreased from 15.41 ± 5.11 at baseline to 9.12 ± 4.57 at 7 days and 6.32 ± 4.77 at 30 days. Mean SCORAD scores similarly declined from 45.89 ± 11.51 at baseline to 29.59 ± 12.63 at 7 days and 21.28 ± 13.93 at 30 days (Table 2).

Repeated-measures ANOVA confirmed a statistically significant effect of time on POEM scores (Mauchly’s W = 0.832, *p* = 0.028; Greenhouse–Geisser corrected: F(1.71, 68.48) = 94.07, *p* < 0.001, partial η^2^ = 0.702) and SCORAD scores (Mauchly’s W = 0.760, *p* = 0.005; Greenhouse–Geisser corrected: F(1.61, 64.50) = 116.08, *p* < 0.001, partial η^2^ = 0.744), indicating very large effect sizes (Table 3a). Bonferroni-corrected pairwise comparisons showed significant differences between all three time point comparisons for both outcomes (all *p* < 0.001), demonstrating that significant improvement occurred during the first 7 days and continued through day 30 (Table 3b).

**Figure 1:**
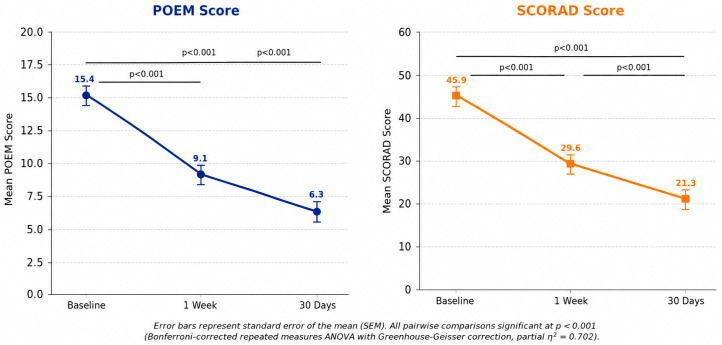
Evolution of POEM and SCORAD scores over 30 days (N = 41).

The longitudinal evolution of both outcome measures is illustrated in Figure 1.

**Figure 2:**
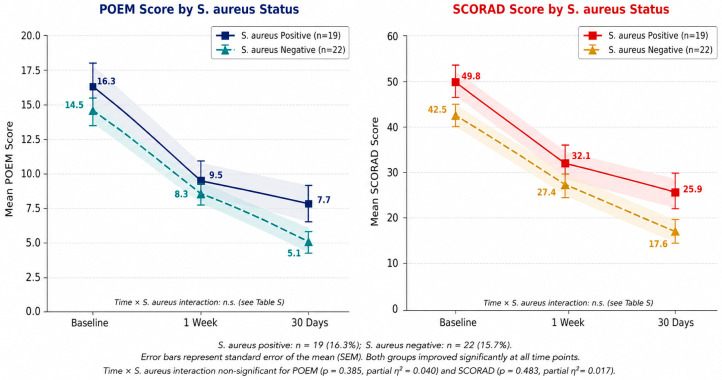
POEM and SCORAD trajectories by Staphylococcus aureus skin colonization status.

Patients with positive *Staphylococcus aureus* cultures tended to have higher POEM and SCORAD scores throughout follow-up than culture-negative patients, although both groups demonstrated progressive improvement (Figure 2). Similarly, patients requiring systemic therapy generally presented with higher disease severity. However, mixed ANOVA demonstrated no significant time × group interaction for either *S. aureus* status (POEM: partial η^2^ = 0.024, *p* = 0.385; SCORAD: partial η^2^ = 0.017, *p* = 0.483) or systemic therapy (both *p* > 0.65), indicating that neither factor significantly modified the trajectory of improvement (Table 4). The between-group differences at 30 days were imprecise (*S. aureus*-positive vs. -negative: POEM +2.55, 95% CI −0.40 to 5.49; SCORAD +8.52, 95% CI 0.03 to 17.02). Because these subgroup analyses were underpowered a priori, they should be considered exploratory.

### 3.3. Predictors of 30-Day Disease Severity

Hierarchical linear regression was subsequently performed to identify independent predictors of POEM and SCORAD scores at 30 days (Table 5). These analyses should be considered exploratory and hypothesis-generating because, with N = 41 and seven candidate predictors, the events-per-variable ratio (approximately 5.9) was below the recommended 10–15 observations per predictor, increasing the risk of model overfitting.

For POEM, the baseline score significantly predicted 30-day severity (Model 1: R^2^ = 0.220, *p* = 0.002), explaining 22.0% of the variance. Model 2 additionally included demographic and perinatal characteristics (age, sex, prematurity, breastfeeding, cesarean section, and in vitro fertilization); however, these variables did not significantly improve model fit (ΔR^2^ = 0.054, *p* = 0.872).

Similarly, baseline SCORAD significantly predicted 30-day outcome (Model 1: R^2^ = 0.310, *p* < 0.001), accounting for 31.0% of the variance. Model 2 additionally included the same demographic and perinatal characteristics; however, these variables did not significantly improve prediction (ΔR^2^ = 0.083, *p* = 0.612). Overall, baseline disease severity was the strongest predictor of 30-day clinical status, whereas the evaluated demographic and perinatal characteristics did not provide additional predictive value in this cohort.

## 4. Discussion

The present study characterized the microbiological profile and short-term clinical evolution of pediatric patients with infected, non-responsive atopic dermatitis managed in routine clinical practice. The principal findings were that *Staphylococcus aureus* was the predominant microorganism isolated from infected lesions, disease severity improved significantly over the 30-day follow-up as reflected by both POEM and SCORAD scores, and baseline disease severity emerged as the strongest predictor of short-term clinical outcomes, whereas demographic and perinatal characteristics did not independently predict disease evolution [22]. These findings provide clinically relevant information regarding the microbiological characteristics and short-term response to individualized management in children with infected, treatment-resistant atopic dermatitis. They should be interpreted in the context of the well-established role of skin microbiota in maintaining epidermal barrier integrity and immune homeostasis, as alterations in microbial composition contribute to the pathogenesis and progression of atopic dermatitis throughout childhood [22,23].

Microbiological evaluation identified *Staphylococcus aureus* as the predominant pathogen, consistent with previous reports describing skin dysbiosis and increased *S. aureus* colonization as common features of more severe or treatment-resistant atopic dermatitis [24,25,26,27,28,29]. Healthy skin harbors a diverse microbial community composed of bacteria, fungi, and viruses, whereas atopic dermatitis is characterized by reduced microbial diversity and predominance of *S. aureus*, which has been associated with disease exacerbation and secondary skin infection [30,31,32]. Although the present study was not designed to investigate the molecular mechanisms underlying skin dysbiosis, the high prevalence of *S. aureus* observed in our cohort supports its clinical relevance in children with complicated atopic dermatitis and reinforces the value of culture-based microbiological assessment in guiding individualized therapeutic management [33,34,35,36]. The predominance of methicillin-sensitive *S. aureus* isolates observed in this cohort may also have practical implications for antimicrobial selection in routine clinical practice, although this observation should be confirmed in larger multicenter studies [37].

The significant clinical improvement observed in our cohort is consistent with current management strategies for complicated atopic dermatitis, which emphasize restoration of skin barrier function together with individualized anti-inflammatory and antimicrobial therapy when clinically indicated [30,31,32,33,34,35,36,37,38]. Although the present study was not designed to evaluate the effectiveness of individual therapeutic agents, the favorable evolution of both POEM and SCORAD scores suggests that individualized management based on clinical presentation and microbiological findings was associated with substantial short-term improvement. The consistent reduction observed in both clinician-assessed (SCORAD) and patient-reported (POEM) outcomes indicates that improvement occurred across complementary dimensions of disease severity, strengthening the overall interpretation of treatment response [39]. Although patients requiring systemic therapy or presenting with *Staphylococcus aureus* colonization tended to have higher disease severity throughout follow-up, neither factor significantly modified the trajectory of clinical improvement. These findings should be interpreted cautiously because the subgroup analyses were exploratory and the study was not powered to detect modest interaction effects, but they suggest that meaningful clinical improvement was observed across the study cohort regardless of baseline subgroup status [40]. Improvement was evident after the first week of treatment and was maintained throughout the 30-day follow-up, supporting the effectiveness of this individualized therapeutic approach in routine clinical practice.

Early-life factors such as mode of delivery, prematurity, and infant microbial colonization have been proposed to influence skin microbiota development and susceptibility to atopic dermatitis [39,40,41,42,43]. Infants born by cesarean section acquire a microbial profile that differs from that of vaginally delivered infants, and several studies have suggested that these early microbial differences may influence immune maturation and subsequent allergic disease risk [40,41]. In our cohort, however, although most children were delivered by caesarean section, neither mode of delivery nor the other evaluated perinatal characteristics independently predicted disease severity at 30 days after adjustment for baseline severity [42]. These findings suggest that, within this cohort of children with established infected, non-responsive atopic dermatitis, baseline clinical severity was a stronger determinant of short-term outcome than the evaluated perinatal factors [43].

Hierarchical regression analyses identified baseline disease severity as the strongest predictor of clinical status at 30 days, whereas prematurity, mode of delivery, breastfeeding, sex, age, and in vitro fertilization did not independently predict short-term clinical outcomes. These findings suggest that the initial clinical presentation may be more informative for predicting short-term response than the evaluated demographic or perinatal characteristics [43,44]. Previous studies have suggested that gestational age and other early-life factors may influence skin microbiota composition and susceptibility to atopic dermatitis; however, the available evidence remains inconsistent [44,45,46,47]. Environmental factors, including ultraviolet radiation, pollution, hygiene practices, and climate, have also been reported to influence skin microbial composition and the clinical course of atopic dermatitis [48,49], while differences between urban and rural environments have been associated with distinct skin microbial communities and variations in AD prevalence [50]. Although these factors may contribute to disease susceptibility and pathogenesis, our findings indicate that they did not independently influence short-term clinical evolution after adjustment for baseline disease severity. From a clinical perspective, these findings suggest that careful assessment of baseline disease severity may be more informative for short-term prognostic evaluation than the evaluated demographic or perinatal characteristics and may help identify children requiring closer clinical monitoring during the initial phase of treatment. Nevertheless, larger prospective studies are warranted to further clarify the prognostic role of these variables.

The study has several strengths. The prospective design, microbiological confirmation of infected lesions, complete follow-up of all enrolled participants, and standardized assessment using two complementary validated outcome measures (POEM and SCORAD) allowed for a comprehensive evaluation of short-term clinical evolution in a well-defined cohort of children with treatment-resistant atopic dermatitis. The integration of microbiological findings with standardized clinical outcome measures also enabled characterization of the relationship between pathogen profile and clinical evolution under routine clinical practice conditions, providing information that may assist clinicians managing similar patient populations.

Several limitations should also be acknowledged. The relatively small sample size limited statistical power, particularly for subgroup and multivariable analyses, and the single-center design limits the generalizability of the findings. Microbiological cultures were performed only in children presenting with clinically infected, non-responsive lesions and therefore do not represent the microbiological profile of all patients with atopic dermatitis. Although all enrolled participants completed the scheduled assessments, the 30-day follow-up allowed for the evaluation of short-term outcomes only and did not permit assessment of long-term disease control, recurrence, or persistence of microbial colonization following treatment. In addition, the absence of a control group precludes conclusions regarding the causal effectiveness of the individualized therapeutic approach. Future multicenter studies with larger cohorts and longer follow-up are warranted to validate these findings and determine their applicability to broader pediatric populations.

## 5. Conclusions

Atopic dermatitis is a complex immune-mediated inflammatory skin disease with heterogeneous clinical presentation and evolution. In this Romanian observational study of 41 pediatric patients with non-responsive atopic dermatitis and infected skin lesions, both POEM and SCORAD scores showed a marked and statistically significant reduction over the 30-day follow-up period. These findings were associated with improved clinician-assessed disease severity and patient-reported symptom burden following individualized management.

The most frequently identified pathogen was *Staphylococcus aureus*, particularly methicillin-sensitive *S. aureus* (MSSA), although other bacterial species were also isolated. Despite the high prevalence of *S. aureus* colonization and the frequent use of topical or systemic antimicrobial therapy, subgroup analyses did not demonstrate a significant effect of systemic treatment or *S. aureus* status on the overall trajectory of clinical improvement.

Baseline disease severity was the strongest predictor of 30-day POEM and SCORAD outcomes, whereas the evaluated demographic and perinatal characteristics did not provide additional predictive value in this cohort. Overall, these findings suggest that baseline clinical severity and microbiological assessment may be useful components of the short-term evaluation of children with infected, non-responsive atopic dermatitis. However, given the observational design and relatively small sample size, larger prospective controlled studies are required to confirm these findings and determine their broader clinical applicability.

## Figures and Tables

**Table 1:** Baseline characteristics of the study population (N = 41).

Characteristic	n (%) or Mean ± SD
Sex	
Male	24 (58.5)
Female	17 (41.5)
Age (months)	39.78 ± 45.64
Median (IQR)	12 (7–60)
Age ≤ 5 years (≤60 months)	31 (75.6)
Age > 5 years (>60 months)	10 (24.4)
Mode of delivery	
Cesarean section	34 (82.9)
Vaginal delivery	7 (17.1)
Premature birth	
Yes	12 (29.3)
No	29 (70.7)
In vitro fertilization	
Yes	5 (12.2)
No	36 (87.8)
Breastfed as a newborn	
Yes	15 (36.6)
No	26 (63.4)
Staphylococcus aureus culture	
Positive	19 (46.3)
Negative	22 (53.7)
Other bacterial cultures	
Positive	14 (34.1)
Negative	27 (65.9)
Local treatment	
Emollients	36 (87.8)
Topical corticosteroids	25 (61.0)
Topical antibiotics	34 (82.9)
Systemic therapy	
Yes	11 (26.8)
No	30 (73.2)
Baseline POEM score	15.41 ± 5.11
Baseline SCORAD score	45.89 ± 11.51

IQR, interquartile range; POEM, Patient-Oriented Eczema Measure; SCORAD, SCORing Atopic Dermatitis.

**Table 2:** Descriptive statistics of the POEM and SCORAD scores across all time points.

Variables	Minimum	Maximum	Mean ± SD
POEM Baseline	7	28	15.41 ± 5.11
POEM 7 days	3	21	9.12 ± 4.57
POEM 30 days	2	24	6.32 ± 4.77
SCORAD Baseline	22.65	78.50	45.89 ± 11.51
SCORAD 7 days	11.25	65.00	29.59 ± 12.63
SCORAD 30 days	7.75	78	21.28 ± 13.93

**Table 3:** (**a**) Omnibus time effect from repeated-measures ANOVA for POEM and SCORAD; (**b**) repeated measures ANOVA with Greenhouse–Geisser correction.

(**a**)							
**Outcome**	**Mauchly’s W**	***p*** **-Value**	**Greenhouse–Geisser ε**	**F (df)**	***p*** **-Value**	**Partial η^2^**	**Observed Power**
POEM	0.832	0.028	0.856	94.07 (1.71, 68.48)	<0.001	0.702	1.000
SCORAD	0.760	0.005	0.806	116.08 (1.61, 64.50)		0.744	
(**b**)							
**Pairwise Comparison**				**Mean Difference** **(95%CI)**			**Adjusted *p*-Value**
POEM Baseline			POEM 7 Days	6.29 (4.91 to 7.68)			<0.001
			POEM 30 Days	9.09 (7.11 to 11.09)			
POEM 7 Days			POEM 30 Days	2.81 (1.14 to 4.47)			
SCORAD Baseline			SCORAD 7 Days	16.30 (13.33 to 19.27)			<0.001
			SCORAD 30 Days	24.61 (19.87 to 29.36)			
SCORAD 7 Days			SCORAD 30 days	8.31 (3.92 to 12.70)			

**Table 4:** Mixed ANOVA examining the effects of systemic therapy and *Staphylococcus aureus* status on POEM and SCORAD trajectories over the 30-day follow-up.

Analysis	Mean Square	F	*p*-Value	Partial η^2^	Observed Power
POEM -Systemic therapy	0.564	0.05	0.931	0.001	0.057
POEM -*S. aureus* status	10.81	0.97	0.385	0.024	0.197
SCORAD -Systemic therapy	25.59	0.368	0.649	0.009	0.102
SCORAD -*S. aureus* status	46.89	0.67	0.483	0.017	0.147

**Table 5:** Hierarchical linear regression models predicting 30-day POEM and SCORAD scores.

Model	R^2^	ΔR^2^	*p*-Value
POEM (Model 1)	0.220	-	0.002
POEM (Model 2)	0.274	0.054	0.872
SCORAD (Model 1)	0.310	-	<0.001
SCORAD (Model 2)	0.393	0.083	0.612

Model 2 adjusted for age, sex, prematurity, breastfeeding, cesarean section, and in vitro fertilization.

## Data Availability

The original contributions presented in this study are included in the article. Further inquiries can be directed to the corresponding authors.

## Abbreviations

The following abbreviations are used in this manuscript:

AD	atopic dermatitis
FA	food allergy
*Staf. aureus*	*Staphylococcus aureus*
AMP	antimicrobial peptides
